# TREK-1 Regulates Cytokine Secretion from Cultured Human Alveolar Epithelial Cells Independently of Cytoskeletal Rearrangements

**DOI:** 10.1371/journal.pone.0126781

**Published:** 2015-05-22

**Authors:** Andreas Schwingshackl, Esra Roan, Bin Teng, Christopher M. Waters

**Affiliations:** 1 Department of Pediatrics, University of Tennessee Health Science Center, Memphis, TN, United States of America; 2 Department of Physiology, University of Tennessee Health Science Center, Memphis, TN, United States of America; 3 Department of Biomedical Engineering, University of Memphis, Memphis, TN, United States of America; 4 Department of Medicine, University of Tennessee Health Science Center, Memphis, TN, United States of America; University of Giessen Lung Center, GERMANY

## Abstract

**Background:**

TREK-1 deficient alveolar epithelial cells (AECs) secrete less IL-6, more MCP-1, and contain less F-actin. Whether these alterations in cytokine secretion and F-actin content are related remains unknown. We now hypothesized that cytokine secretion from TREK-1-deficient AECs was regulated by cytoskeletal rearrangements.

**Methods:**

We determined F-actin and α-tubulin contents of control, TREK-1-deficient and TREK-1-overexpressing human A549 cells by confocal microscopy and western blotting, and measured IL-6 and MCP-1 levels using real-time PCR and ELISA.

**Results:**

Cytochalasin D decreased the F-actin content of control cells. Jasplakinolide increased the F-actin content of TREK-1 deficient cells, similar to the effect of TREK-1 overexpression in control cells. Treatment of control and TREK-1 deficient cells with TNF-α, a strong stimulus for IL-6 and MCP-1 secretion, had no effect on F-actin structures. The combination of TNF-α+cytochalasin D or TNF-α+jasplakinolide had no additional effect on the F-actin content or architecture when compared to cytochalasin D or jasplakinolide alone. Although TREK-1 deficient AECs contained less F-actin at baseline, quantified biochemically, they contained more α-tubulin. Exposure to nocodazole disrupted α-tubulin filaments in control and TREK-1 deficient cells, but left the overall amount of α-tubulin unchanged. Although TNF-α had no effect on the F-actin or α-tubulin contents, it increased IL-6 and MCP-1 production and secretion from control and TREK-1 deficient cells. IL-6 and MCP-1 secretions from control and TREK-1 deficient cells after TNF-α+jasplakinolide or TNF-α+nocodazole treatment was similar to the effect of TNF-α alone. Interestingly, cytochalasin D decreased TNF-α-induced IL-6 but not MCP-1 secretion from control but not TREK-1 deficient cells.

**Conclusion:**

Although cytochalasin D, jasplakinolide and nocodazole altered the F-actin and α-tubulin structures of control and TREK-1 deficient AEC, the changes in cytokine secretion from TREK-1 deficient cells cannot be explained by cytoskeletal rearrangements in these cells.

## Introduction

We previously identified the 2-pore domain potassium (K2P) channel TREK-1 as an important molecule in the regulation of alveolar epithelial cell (AEC) cytokine secretion[1–3], cell detachment[4] and proliferation[1]. Our data revealed that TREK-1 deficient AECs secrete lower amounts of IL-6 but increased amounts of MCP-1 upon TNF-α stimulation[1–3]. Furthermore, in an *in vivo* model of Acute Lung Injury (ALI) we recently found that TREK-1 deficiency led to increased lung damage and AEC apoptosis but decreased BAL cytokine levels[5]. In a separate study, we recently reported that TREK-1 deficient AECs contained lower amounts of F-actin and these cells appeared more resistant to stretch-induced injury[4].

Based on these results, the main goal of this study was to determine whether the alterations in cytokine secretion from TREK-1 deficient AECs were caused by changes in the cytoskeletal filament content and organization observed in these cells. We hypothesized that the impaired IL-6 secretion from TREK-1 deficient AECs was related to the decreased F-actin content of these cells, whereas the increased secretion of MCP-1 was unrelated to cytoskeletal derangements.

In general, inflammatory mediators such as cytokines and other soluble molecules are thought to be packaged in the Golgi apparatus into secretory vesicles, or so-called Secretory Carrier Membrane Proteins (SCAMPs)[6], and transported to the correct location at the plasma membrane along a cytoskeletal network of F-actin fibers and microtubules[7–12]. This phenomenon is best described in inflammatory cells and is commonly known as compound exocytosis[13,14]. Unfortunately, little is known about the molecular mechanisms regulating mediator secretion from AECs and their contribution to lung inflammation and lung injury.

Nevertheless, the cytoskeleton appears to play an active role in AECs in the secretion of both soluble inflammatory mediators such as cytokines and chemokines[15,16] as well as reactive oxygen[17] and nitrogen species[18]. Specifically, in AECs a role for F-actin and microtubules has been proposed for the secretion of TNF-α, IL-6, MCP-1, IL-8[16,19–21], surfactant[22] and fibrinogen[23]. However, most of these studies were conducted in infectious models of lung inflammation, and the authors often attributed the F-actin-mediated changes in cytokine secretion to a decreased ability of AECs to engulf bacteria, which subsequently resulted in decreased cytokine production[21,24,25]. To the best of our knowledge, the relationship between potassium channel expression, regulation of cytoskeletal structures, and inflammatory mediator secretion from AECs has never been studied. Here we report that in AECs TREK-1 regulates the content and architecture of cytoskeletal filaments, but these changes do not affect the production or secretion of IL-6 or MCP-1.

## Materials and Methods

### Cell culture

Human A549 AECs were purchased from the American Type Culture Collection (ATCC, Manassas, VA). Cells were cultured in DMEM (Gibco, Carlsbad, CA) supplemented with 10% FBS (Gibco), 1% Penicillin/Streptomycin (Gibco), 20mM HEPES (Sigma Aldrich, St. Louis, MO), and 2mM L-Glutamine (Gibco). A stable TREK-1 deficient A549 cell line and a control cell line transfected with a scrambled shRNA were created as previously described[3].

A stable TREK-1 over-expressing A549 cell line was created as described previously[2] using an Origene TrueORF Gold cDNA Clones and Precision Shuttle Vector system (cat#RC210180) by following to the manufacturer’s instructions. Details of the pCMV6-Entry vector containing a DDK-tag for detection are available on the Origene website (www.origene.com/cdna/trueorf/destinationvector.mspx). Briefly, 3x10^5^ cells were grown in 6 well plates prior to transfection until cells reached 60–70% confluence in DMEM medium supplemented with 10% FBS, 20mM HEPES and 2mM L-Glutamine. Cells were transfected with the DNA probe provided by the manufacturer using the Turbofectin 8.0 transfection system and incubated for 24 hours at 37°C. To select for positively transfected cells, cells were cultured in T75 flasks in DMEM medium (10% FBS, 1% Penicillin/Streptomycin, 20mM HEPES and 2mM L-Glutamine) supplemented with 0.5 mg/mL G418. As a control, non-transfected A549 cells were cultured in parallel under the same conditions. TREK-1 over-expression was confirmed by Western Blot and real time PCR[2].

Trek-1 deficient and control cells were incubated in the presence or absence of TNF-α (5 ng/ml), jasplakinolide (0.05 μM, Sigma), cytochalasin D (0.1 μM, Sigma), or nocodazole (0.1μM, Sigma). Cell toxicity was monitored by trypan blue staining and determination of total intracellular protein concentrations using the Bradford assay. We found >90% cell viability under all conditions, as in our previous studies[2,3].

### Confocal Microscopy

Cells were grown to 80–90% confluence and then fixed with 4% paraformaldehyde for 5 min at 4°C, permeabilized with 0.5% Triton X-100 for 10 min, and then blocked with 2% BSA in PBS for 30 min. The cells were then incubated with Rhodamine Phalloidin (1:150, Cytoskeleton, CO) for F-actin staining or an anti-α-tubulin Alexa 488 antibody (1:200, Invitrogen) for 30 min at room temperature. Nuclear staining was obtained using Fluoro Gel II mounting medium containing DAPI (Electron Microscopy Sciences, Hatfield, PA). Images were acquired using a Zeiss 710 confocal imaging system. Emitted fluorescence was collected using a 60x magnification objective lens (NA 1.4 Oil), and the images were recorded using Zen 2009 Light Edition software (Zeiss).

### G- and F-actin assay by Western blot

G- and F-actin fractions were collected as previously described[4]. Briefly, cells were seeded in 6 well culture plates (0.3x10^6^ cells/well) in triplicate and grown to >90% confluence. Cells were washed twice with cold PBS and then lysed in the following solution for 5 min on ice: 1% Triton X-100, 20 mM Tris, 5 mM EGTA, 20 mM NaFl, 25 mM Na pyrophosphate, containing a protease inhibitor cocktail (Roche, Burlington, NC). G-actin containing supernatants were collected and total protein concentrations were determined using the Quick Start Bradford (BioRad, Hercules, CA). Thereafter, F-actin was extracted by adding the following solution: 1% Triton X-100, 20 mM Tris, 5 mM EGTA, 20 mM NaFl, 25 mM Na pyrophosphate, containing a protease inhibitor cocktail (Roche, Burlington, NC) and 5% SDS and 5% deoxycholic acid. After 5 min, samples containing F-actin were removed from the wells using a cell scraper and the samples were centrifuged at 17,000 x g for 20 min at 4°C. To determine the relative amounts of G- and F-actin, we performed western blot experiments loading equal sample volumes per lane and blotted with an antibody against actin (1:1000, Cytoskeleton, CO). GAPDH (1:2000, Cell Signaling) was used as an internal loading control for G-actin. Since F-actin filaments remain attached to the substrate after removal of the G-actin fraction with all other cytosolic components, F-actin can only be normalized to G-actin, but not to cytosolic proteins such as GAPDH or β–actin. Therefore, G-actin concentrations were normalized to GAPDH and data were then expressed as F-actin to total actin (G+F-actin) ratios. Band densities for α-tubulin were normalized to GAPDH. All band densitometry analyses to determine relative quantities of proteins were performed using ImageJ 1.42 software for Windows.

### Gene expression by Real-Time PCR

Total RNA was isolated from 2x10^6^ control or TREK-1 deficient A549 cells using a High Pure RNA Isolation Kit (Roche Applied Science, Mannheim, Germany) according to the manufacturer’s instructions. Single-stranded DNA was synthesized from 1 μg total RNA and Reverse Transcription PCR was performed using a High Capacity cDNA Reverse Transcription kit (Applied Biosystems, CA) according to the manufacturer’s instructions. Real-Time PCR was performed using a TaqMan Gene Expression assay (Roche). Primer sets for human IL-6 were purchased from Cell Signaling and primer sets for MCP-1 were purchased from IDT as previously described[2,3]. In preliminary experiments we confirmed that HGPRT levels were unchanged between control and TREK-1 deficient A549 cells and, therefore, IL-6 and MCP-1 mRNA levels were normalized to HGPRT expression. All experiments were repeated 4 times and each sample was run in triplicates.

### IL-6 and MCP-1 ELISA measurements

IL-6 and MCP-1 secretion in A549 cell supernatants was measured as previously described[2,3]. Briefly, 1x10^5^ control, TREK-1 deficient or overexpressing cells were seeded in 12-well culture plates and grown to 80–90% confluence. Cells were then incubated in complete culture medium in the presence or absence of TNF-α, jasplakinolide (0.05 μM), cytochalasin D (0.1 μM), or nocodazole (0.1 μM) for 2 or 6 hours at 37°C. Total intracellular protein concentrations were measured in each experiment using the Bradford assay and remained consistent under all experimental conditions, suggesting that there was no non-specific leakage of intracellular proteins. Cytokine concentrations were determined from sample supernatants using species-specific IL-6 and MCP-1 ELISA kits (BD Bioscience OptEIA following the manufacturer’s instructions.

### Statistical analysis

All values were expressed as mean ± SEM and statistical analysis was performed using Student’s t-test or ANOVA. All statistical analyses were performed using SigmaStat 3.5 software and a p-value of p<0.05 was considered significant.

## Results

### Regulation of F-actin and α-tubulin filaments in control, TREK-1 deficient and TREK-1 overexpressing AECs

**Fig 1:**
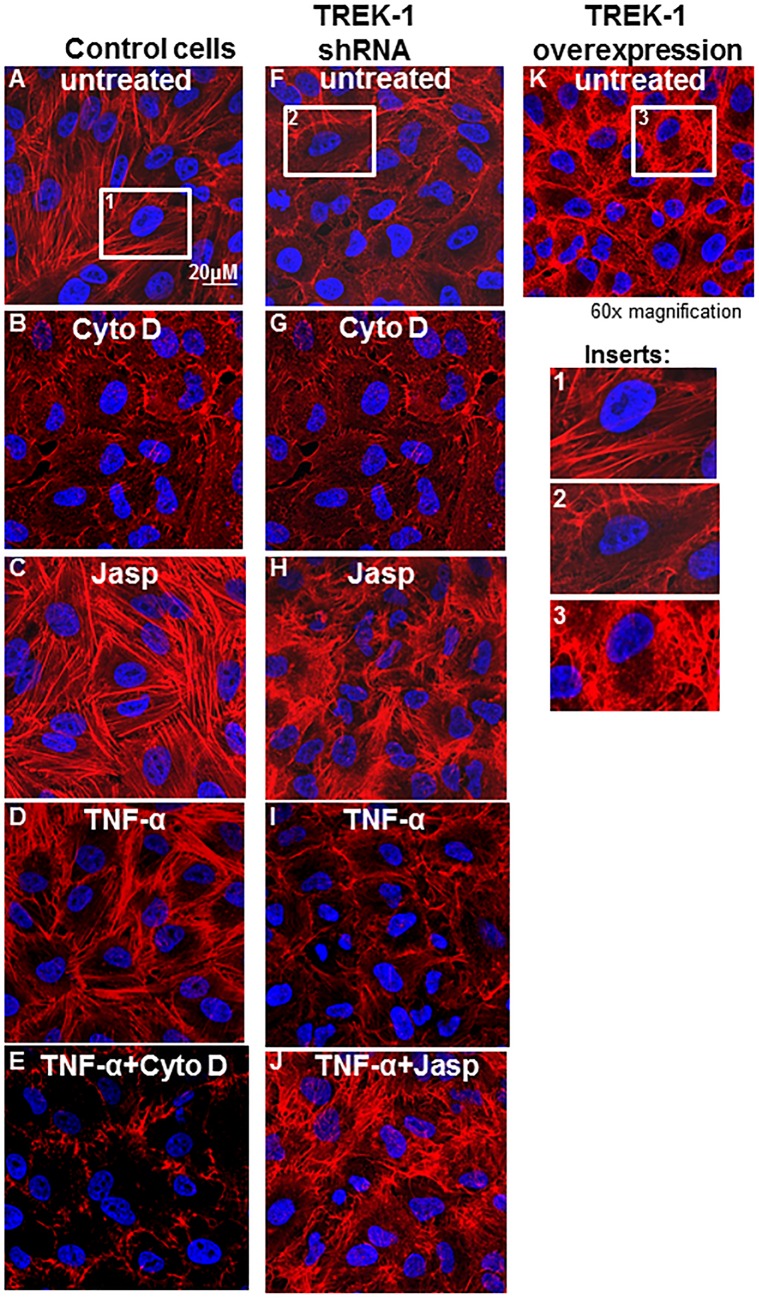
Regulation of F-actin architecture in control, TREK-1 deficient and TREK-1 overexpressing cells. Confocal images using rhodamine-phalloidin staining (red) for F-actin filaments in untreated cells and after 6 hours of treatment with cytochalasin D (0.1 μM), jasplakinolide (0.05 μM) and TNF-α (5 ng/mL), or the combination of TNF-α+cytochalasin D or TNF-α+jasplakinolide. Inserts 1, 2 and 3 show the F-actin structures of a single control, TREK-1 shRNA or TREK-1 overexpressing cell, respectively. Nuclei are stained with DAPI (blue); images are representative of 4 separate experiments.

To test the hypothesis that TREK-1-mediated changes in cytoskeletal structures regulate cytokine secretion from AECs, we manipulated the architecture of F-actin filaments with cytochalasin D (a potent inhibitor of actin polymerization[26,27]), jasplakinolide (a drug known to promote and stabilize actin polymerization[28,29]), TNF-α (a strong stimulus for cytokine secretion from AECs[2,3]), and by overexpressing TREK-1 protein in AECs (Fig 1).

Treatment of AECs with cytochalasin D for 6 hours disrupted F-actin filaments in control (Fig 1B) and TREK-1 deficient cells (Fig 1G), whereas jasplakinolide increased the F-actin content in both cell types (Fig 1C and 1H). Similarly, TREK-1 overexpression also increased the F-actin content in control cells (Fig 1K). TREK-1 deficient cells contained lower amounts of F-actin at baseline[4] (Fig 1F). Treatment of control and TREK-1 deficient cells with TNF-α had no effect on F-actin filaments when compared to untreated cells (Fig 1D and 1I), and the combination of TNF-α+cytochalasin D or TNF-α+jasplakinolide had no additional effect on F-actin filaments when compared with cytochalasin D or jasplakinolide alone, respectively. To observe time-dependent changes in cytoskeletal remodeling, we also exposed AECs to these drugs for 2 hours instead of 6 hours and found similar results (data not shown).

**Fig 2:**
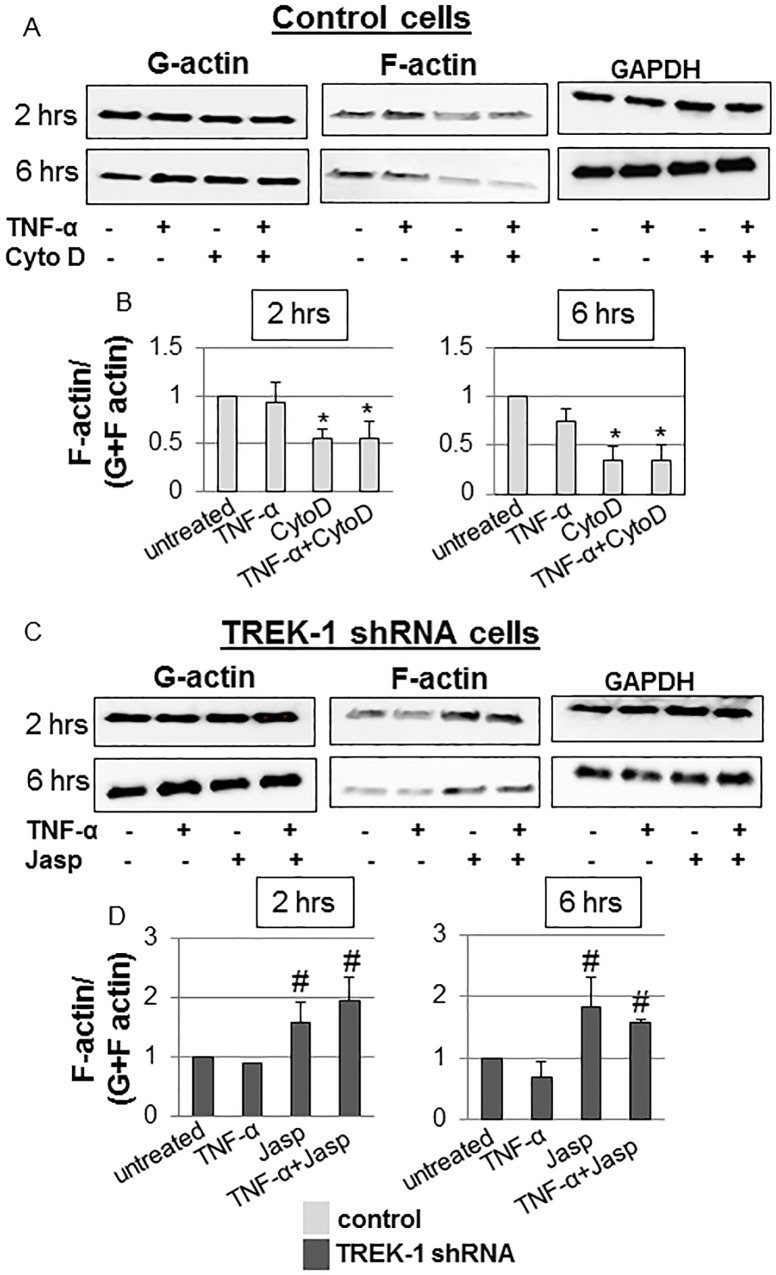
Quantification of normalized F-actin contents in control and TREK-1 deficient cells. Representative western blot experiments and densitometry analysis of cytochalasin D-treated control (Fig 2A and Fig 2B) and jasplakinolide-treated TREK-1 deficient cells (Fig 2C and Fig 2D) in the presence or absence of TNF-α. Importantly, only soluble G-actin can be normalized to GAPDH since F-actin was collected after all soluble components (including intracellular GAPDH) were removed in the G-actin sample. However, G-actin, F-actin and GAPDH samples for the respective time points were run and analyzed from the same gel; the blots are separated in the figure to conserve space and to facilitate comparison. *compared to untreated control or #TREK-1 deficient cells at the 2 and 6 hour time points, respectively. n = 5.

Densitometry quantification of F-actin to total (G+F-actin) actin ratios revealed a decrease in the F/total actin content in control cells after 2 and 6 hours of cytochalasin D treatment by 45% and 66%, respectively (Fig 2A and 2B). Exposure of control cells to TNF-α+cytochalasin D for 2 or 6 hours had no further effect on the F/total actin content when compared to cytochalasin D treatment alone. Exposure of TREK-1 deficient cells to 2 or 6 hours of jasplakinolide increased the F/total actin content by 57% and 85%, respectively, but the combination of TNF-α+jasplakinolide had no further effect on the F/total actin content when compared to jasplakinolide alone (Fig 2C and 2D).

**Fig 3:**
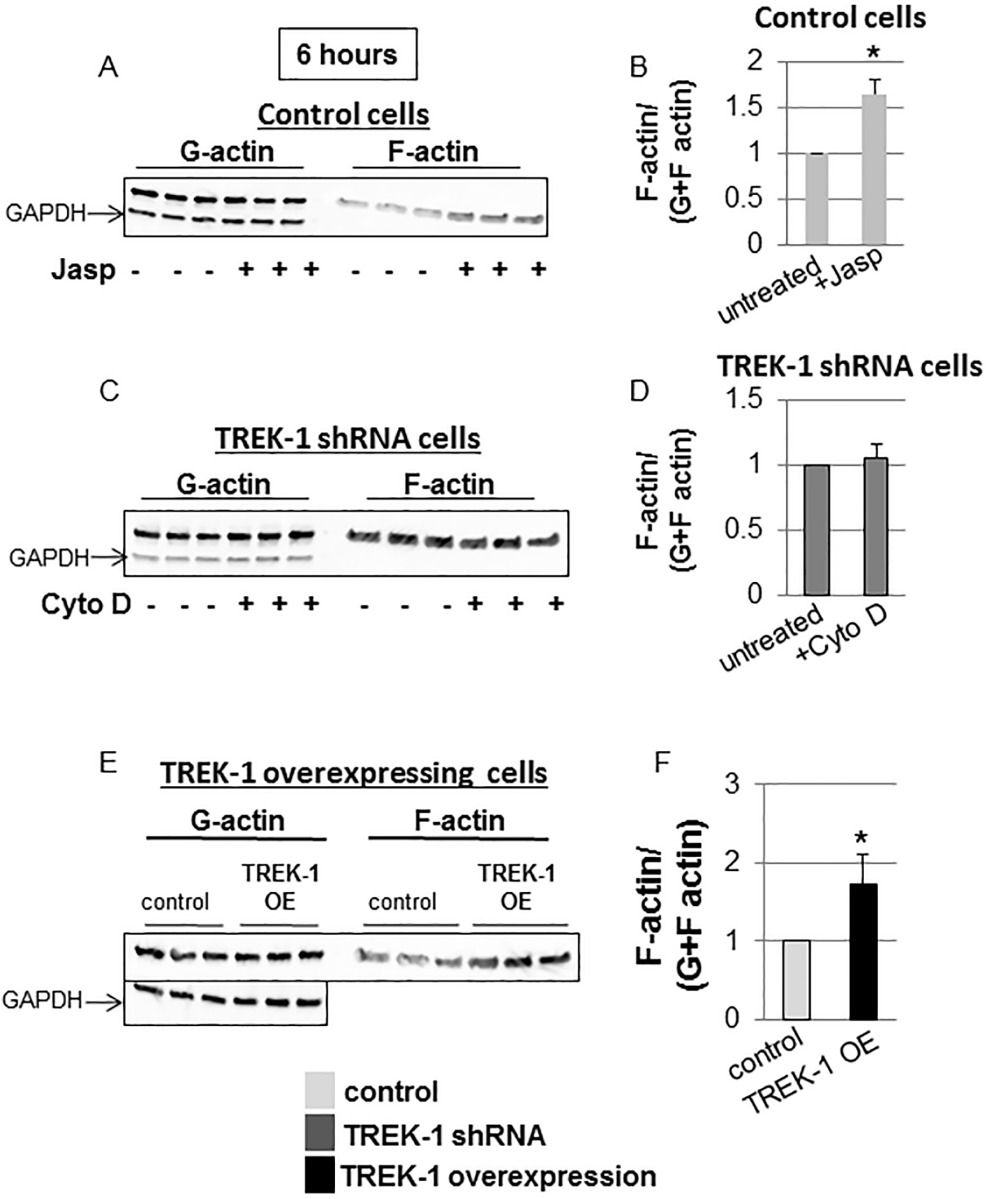
Normalized F-actin contents in control, TREK-1 deficient and TREK-1 overexpressing cells. Representative Western blot experiments and densitometry analysis showing the effects of jasplakinolide on control cells (Fig 3A and Fig 3B) and of cytochalasin D on TREK-1 deficient cells (Fig 3C and Fig 3D). Fig 3E shows an increase in the normalized F-actin content in TREK-1 overexpressing cells. Each lane represents a separate experiment. GAPDH was used as a loading control. *compared to untreated control cells. n = 5.

Furthermore, exposure of control cells to jasplakinolide increased the F/total actin content by 64% (Fig 3A and 3B), whereas cytochalasin D had no further effect on the F/total actin content in TREK-1 deficient cells (Fig 3C and 3D). Similar to jasplakinolide-treated cells, TREK-1 overexpression increased the F/total actin content (73% increase over control; Fig 3E and 3F).

**Fig 4:**
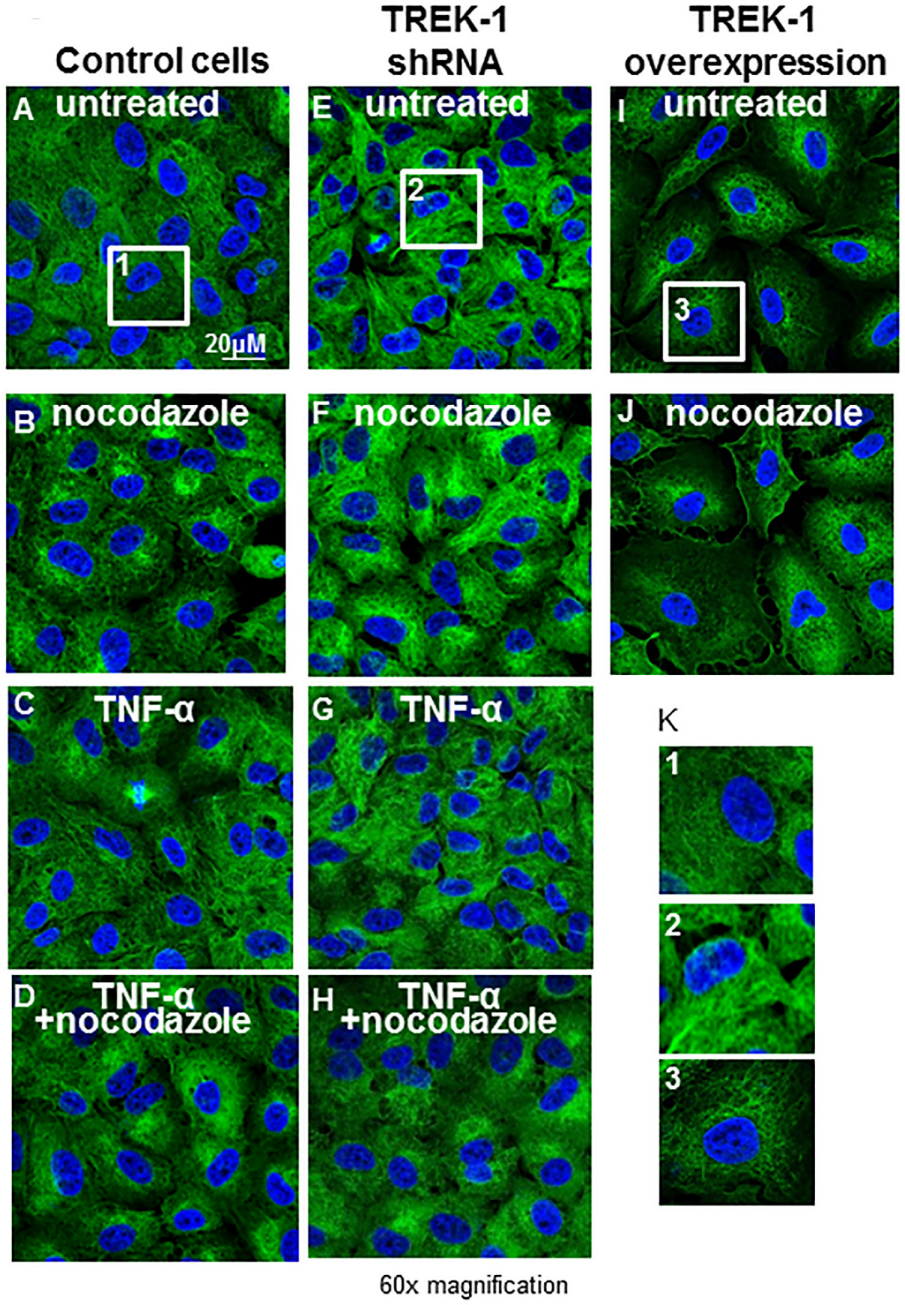
Confocal images of α-tubulin contents and architecture. Confocal images of α-tubulin staining (green) in control, TREK-1 deficient and TREK-1 overexpressing cells in the presence or absence of nocodazole, TNF-α, or the combination of TNF-α+nocodazole for 6 hours. Representative images of 4 separate experiments.

**Fig 5:**
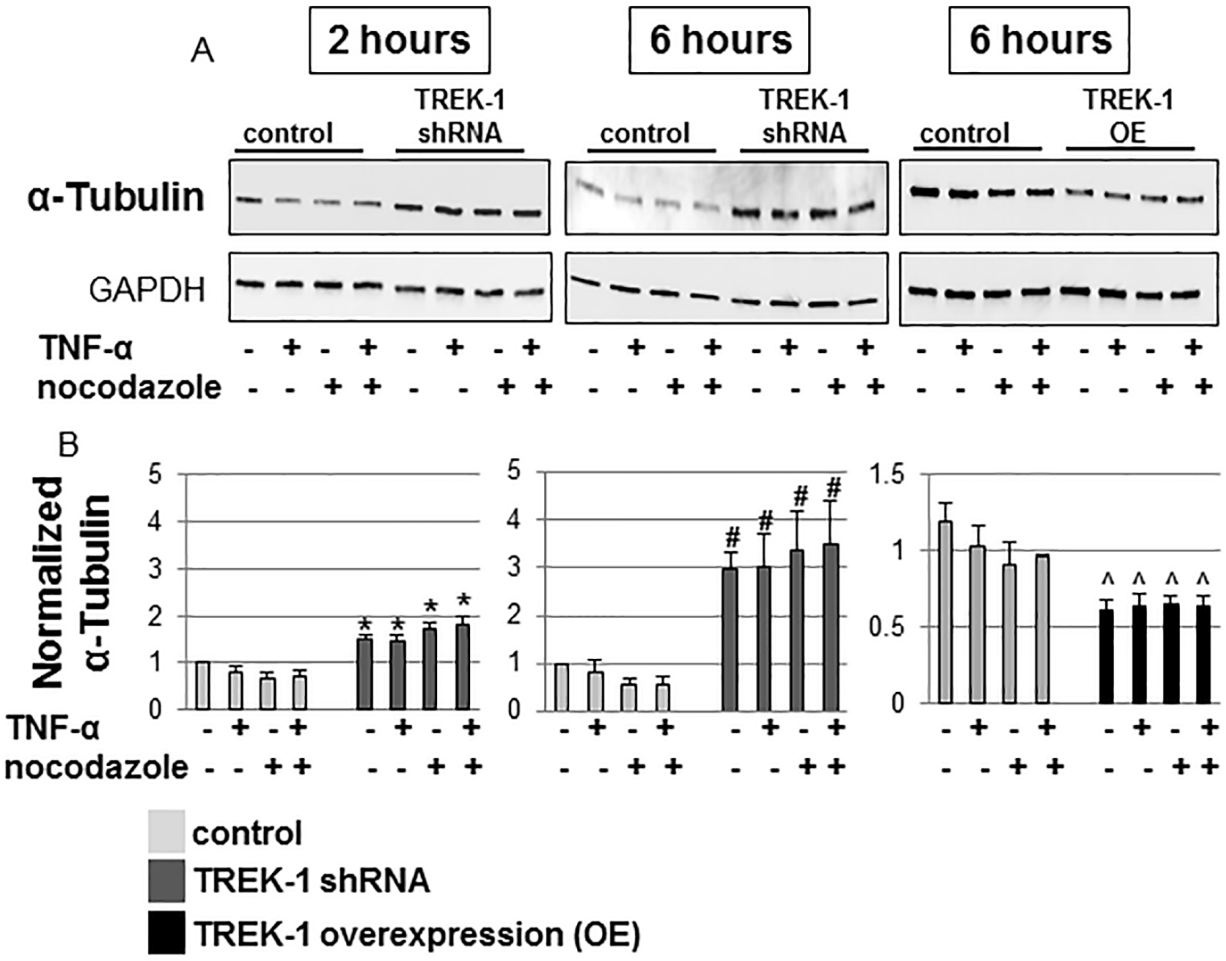
Quantification of the α-tubulin content. Representative Western Blot experiment and densitometric analysis of untreated, nocodazole-treated and TNF-α-treated control and TREK-1 deficient cells. *compared to untreated control cells at the 2 hour time point, #compared to untreated control cells at the 6 hour time point. n = 5.

Interestingly, at baseline, TREK-1 deficient cells contained increased amounts of α-tubulin when compared to control cells as shown by confocal microscopy (Fig 4) and Western blot experiments (Fig 5). Nocodazole disrupted α-tubulin filaments and resulted in perinuclear accumulation of α-tubulin filaments in both control and TREK-1 deficient cells (Fig 4). Treatment of control and TREK-1 deficient cells with TNF-α had no effect on α-tubulin filaments when compared to untreated cells, and the combination of TNF-α+nocodazole had no additional effect on α-tubulin filaments when compared with nocodazole alone. These findings were confirmed by densitometry analysis of 5 separate experiments (Fig 5).

In contrast to TREK-1 deficient cells, TREK-1 overexpressing cells contained lower amounts of α-tubulin as shown by confocal microscopy (Fig 4) and by Western blot (Fig 5). GAPDH immunoblots confirmed equal loading conditions.

### The alterations in IL-6 and MCP-1 secretion from TREK-1 deficient cells were not caused by cytoskeletal derangements

**Fig 6:**
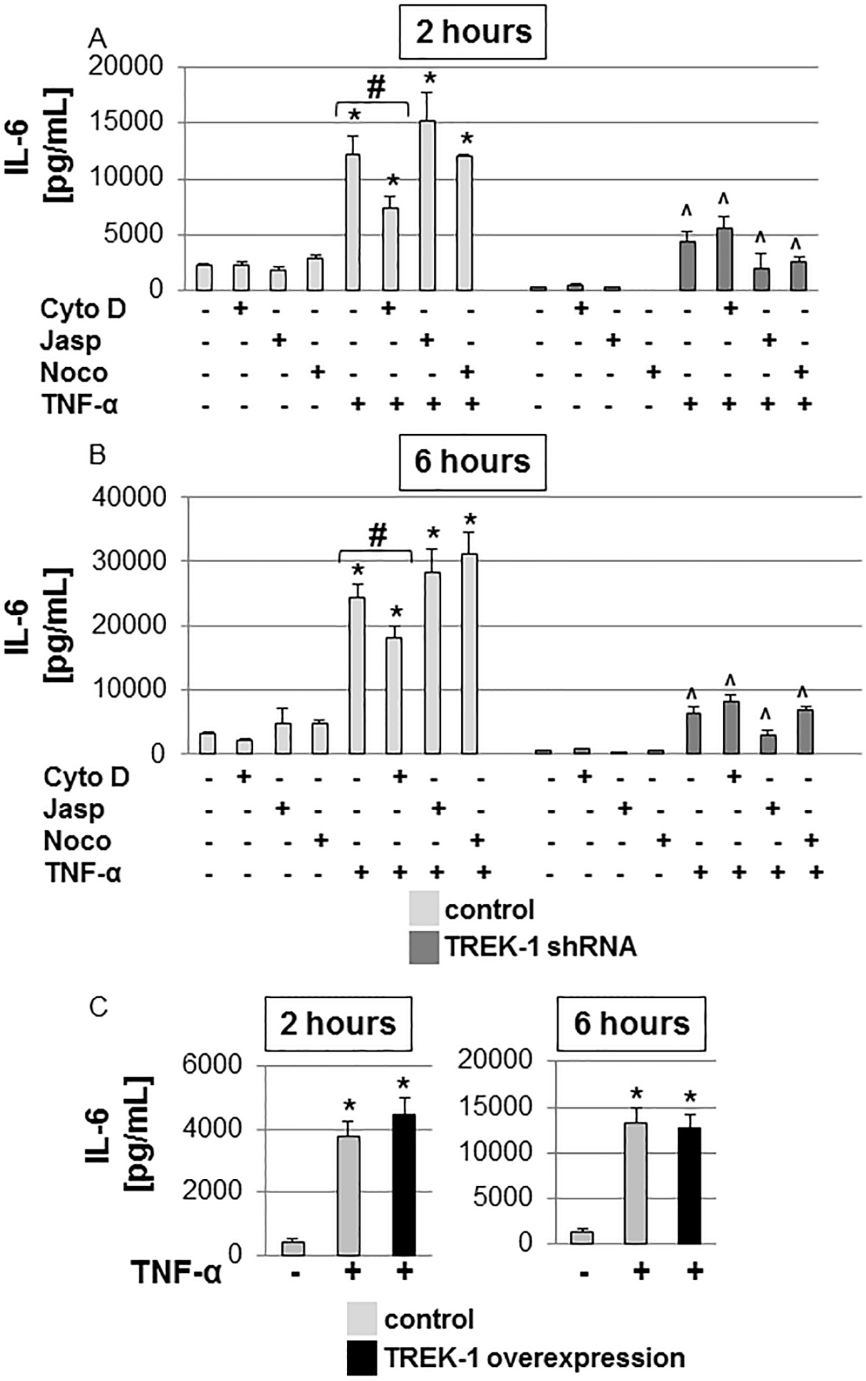
IL-6 secretion was not affected by alterations in F-actin or α-tubulin structures. IL-6 secretion from control and TREK-1 deficient cells was measured after exposure to cytochalasin D (Cyto D), jasplakinolide (Jasp), nocodazole (noco) with or without TNF-α for 2 (Fig 6A) or 6 hours (Fig 6B). TREK-1 overexpression had no additional effect on TNF-α-induced IL-6 secretion Fig 6C). *compared to untreated control cells; ^compared to untreated TREK-1 deficient cells; #TNF-α-stimulated control cells with and without cytochalasin D. n = 6–9.

Exposure of control cells to TNF-α for 2 and 6 hours resulted in a 4.3-fold and 7.8-fold increase in IL-6 secretion, respectively, when compared to untreated cells, while TREK-1 deficient cells released much lower amounts of IL-6 both at baseline and after TNF-α stimulation (Fig 6A and 6B)[3]. Neither jasplakinolide nor nocodazole altered baseline or TNF-α-induced IL-6 secretion from control or TREK-1 deficient cells, respectively (Fig 6A and 6B). However, cytochalasin D decreased IL-6 secretion from control but not TREK-1 deficient cells at both time points (Fig 6A and 6B). In the presence of TNF-α, the inhibitory effect of cytochalasin D on IL-6 release from control cells was no longer observed at either time point.

Exposure of TREK-1 overexpressing cells to TNF-α for 2 or 6 hours caused no further increase in IL-6 secretion when compared to untreated controls (Fig 6C).

**Fig 7:**
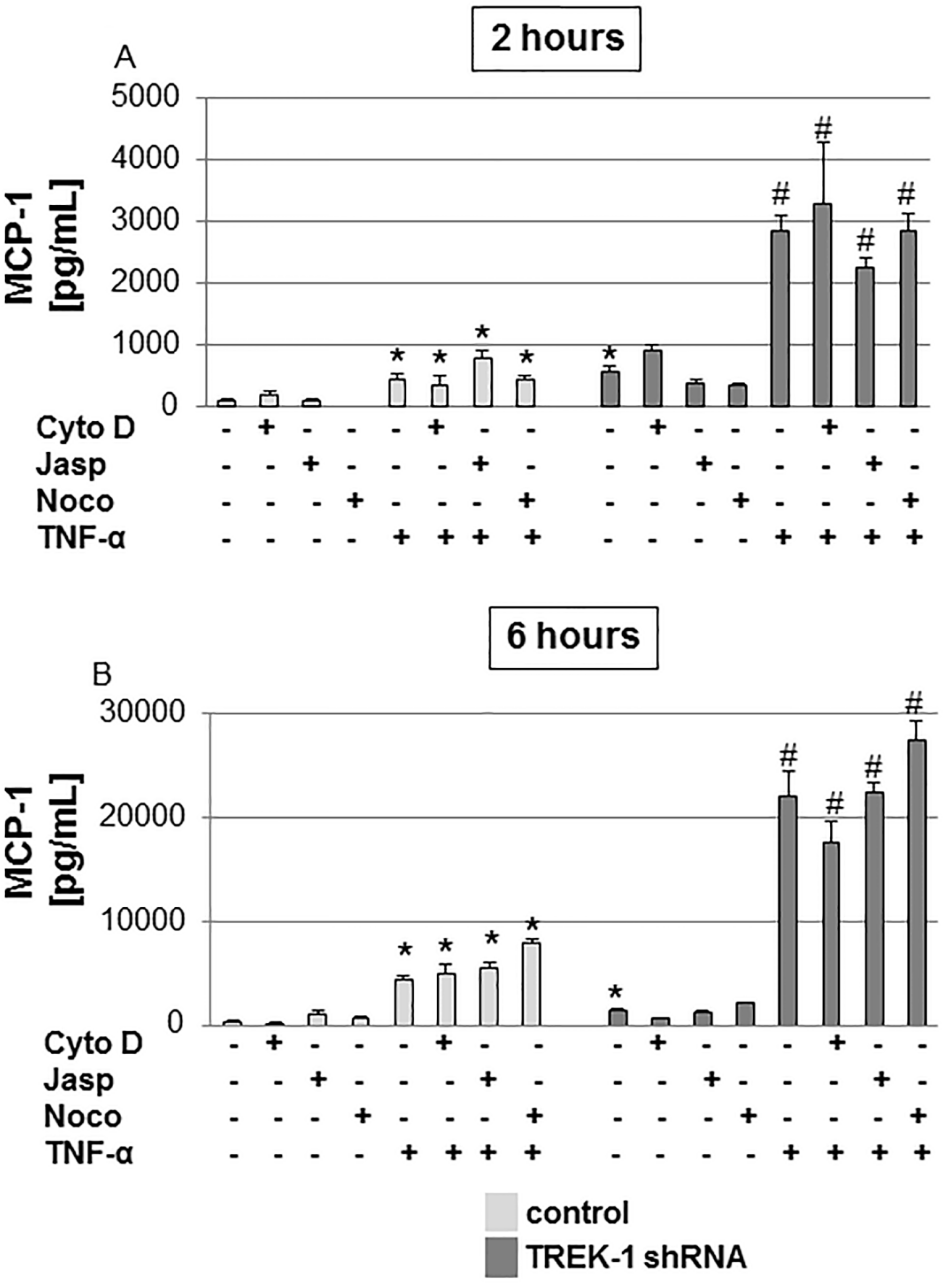
MCP-1 secretion was not affected by alterations in F-actin or α-tubulin structures. MCP-1 secretion from control and TREK-1 deficient cells was measured after exposure to cytochalasin D (Cyto D), jasplakinolide (Jasp), nocodazole (noco) with or without TNF-α for 2 (Fig 7A) or 6 hours (Fig 7B). *compared to untreated control cells; #compared to untreated TREK-1 deficient cells. n = 6–9.

As noted previously[2], at baseline and after TNF-α stimulation, TREK-1 deficient cells secreted increased amounts of MCP-1 when compared to control cells (Fig 7A and Fig 7B). After 2 and 6 hours of TNF-α stimulation, MCP-1 secretion was increased by 6.7-fold and 15-fold, respectively, when compared to untreated TREK-1 deficient cells (Fig 7A and Fig 7B). Cytochalasin D, jasplakinolide and nocodazole had no effect on baseline or TNF-α-induced MCP-1 secretion from control or TREK-1 deficient cells.

Therefore, it is unlikely that the alterations in IL-6 and MCP-1 secretion from TREK-1 deficient cells were caused by the cytoskeletal derangements observed in these cells.

### Cytoskeletal derangements did not cause alterations in IL-6 and MCP-1 gene expression in TREK-1 deficient cells

**Fig 8:**
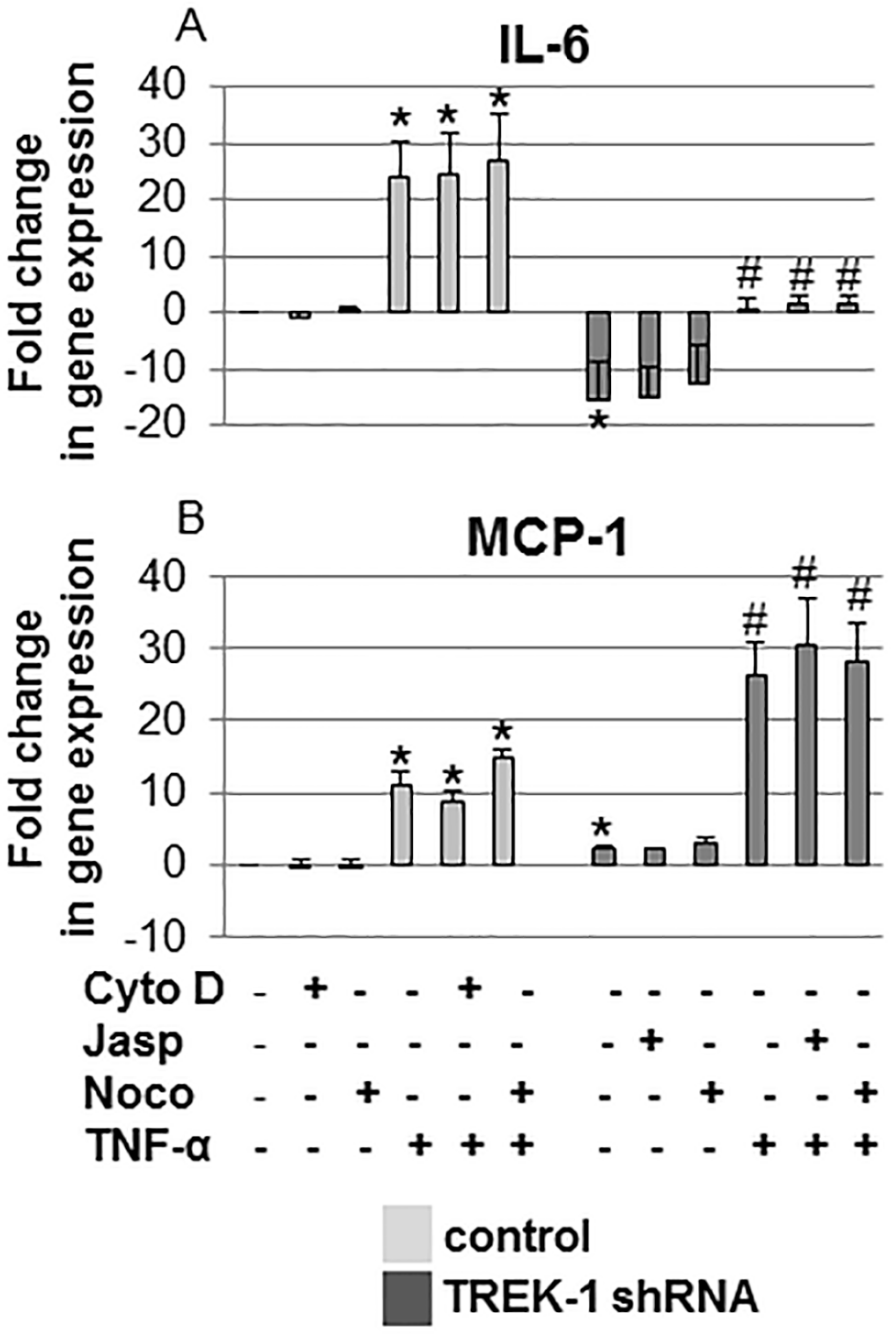
IL-6 and MCP-1 gene expression was not affected by alterations in F-actin or α-tubulin structures. IL-6 (Fig 8A) and MCP-1 (Fig 8B) mRNA expression was determined by quantitative real-time PCR in control and TREK-1 deficient cells exposed to 6 hours of cytochalasin D (Cyto D), jasplakinolide (Jasp) or nocodazole (Noco) in the presence or absence of TNF-α. Data were normalized to HGPRT expression and are expressed as fold-change in gene expression from untreated control cells. *compared to untreated control cells. #compared to untreated TREK-1 deficient cells. A 2-fold change in gene expression was considered significant. n = 4.

To determine whether in TREK-1 deficient AECs cytoskeletal derangements caused alterations in cytokine secretion by affecting IL-6 and MCP-1 gene expression, we investigated the effects of cytochalasin D, jasplakinolide and nocodazole on IL-6 and MCP-1 mRNA levels using real-time PCR (Fig 8A and 8B). At baseline, untreated TREK-1 deficient cells expressed lower amounts of IL-6 mRNA but higher amounts of MCP-1 mRNA than control cells. Treatment with cytochalasin D, jasplakinolide or nocodazole did not affect baseline IL-6 or MCP-1 gene expression in control or TREK-1 deficient cells. Similarly, treatment of control and TREK-1 deficient cells with TNF-α or the combination of TNF-α+cytochalasin D, TNF-α+jasplakinolide or TNF-α+nocodazole had no additional effect on IL-6 and MCP-1 gene expression over TNF-α alone. Although TREK-1 deficient cells contained lower amounts of IL-6 mRNA, the overall fold increase in IL-6 gene expression induced by TNF-α was similar between control and TREK-1 deficient cells when compared to their respective untreated controls. In contrast, MCP-1 mRNA levels increased more in TREK-1 deficient cells that in control cells after TNF-α stimulation.

## Discussion

In this study we showed for the first time that epithelial production and secretion of IL-6 and MCP-1, two important cytokines in the development of Acute Lung Injury (ALI) and Acute Respiratory Distress Syndrome (ARDS)[30,31], can occur independently of F-actin and α-tubulin cytoskeletal rearrangements. Importantly, not only do TREK-1 deficient AECs contain decreased amounts of F-actin[4], but we now report that they also contain increased amounts of α-tubulin. The main goal of this study was to determine whether the distinct cytokine secretion patterns from TREK-1 deficient AECs were caused by changes in the cytoskeletal filament content and structure of these cells.

We are still in the early stages of understanding the role for TREK-1 in the regulation of AEC activation and its contribution to the development of ALI/ARDS[1–3,5]. In previous studies we found that NF_k_B, p38 kinase and JNK kinase are unlikely involved in TREK-1-mediated cytokine secretion, whereas PKCθ appeared to regulate IL-6 but not MCP-1 secretion[2,3]. However, the role of the cytoskeleton in epithelial mediator secretion remains poorly understood and is of particular interest in light of the astonishing cytoskeletal changes observed in TREK-1 deficient cells at baseline. Importantly, a role for the cytoskeleton in cytokine secretion from secretory cells has been described in several studies. Various modes of exocytosis and mediator release, including compound exocytosis, “kiss-and-run” exocytosis, and “full-collapse-fusion” exocytosis, have been described in different secretory cells[13,14,32,33]. All these mechanisms imply a role for cytoskeletal structures in the transport of mediator-containing vesicles from the Golgi apparatus to the plasma membrane[34–36]. For instance, activation of pancreatic β-cells resulted in F-actin reorganization promoting the transport of insulin-containing granules to the plasma membrane[36], and in eosinophils toxic granules translocated to the plasma membrane during apoptosis via F-actin rearrangements[17]. Interestingly, Bengtsson et al. showed in neutrophils that disruption of F-actin filaments with cytochalasin D resulted in increased neutrophil degranulation whereas accumulation of F-actin filaments with tertracaine inhibited mediator secretion[37]. The authors explained these findings by accumulation of F-actin filaments in the cell periphery thereby obstructing secretory granules from fusing with the plasma membrane. Our data showed that neither disruption nor stabilization of F-actin fibers altered TNF-α-induced production or secretion of IL-6 and MCP-1 from control and TREK-1 deficient AECs, respectively. Therefore, the intrinsic scarcity of F-actin fibers present in TREK-1 deficient cells is unlikely the cause for the decreased amounts of IL-6 secreted from these cells.

A role not only for F-actin but also for microtubules in mediator secretion has been described in NK cells, where F-actin stabilization with jasplakinolide trapped lytic granules within an F-actin mesh[38]. Other groups proposed that in activated NK cells specific areas within this F-actin mesh opened and created gaps large enough for granules to penetrate and get secreted[39,40]. Similar mechanisms appear to exist in CD4+ T cells where F-actin and microtubule rearrangements cleared the path for secretory granules to reach the plasma membrane[41]. In contrast, in monocytes, secretion of matrix metalloproteinase-9 was inhibited after disruption of F-actin filaments and microtubules with cytochalasin B and nocodazole[42]. Similar to monocytes, mediator secretion from antigen-stimulated mast cells[43] and from neuronal cells[44] was impaired after disruption of microtubules with colchicine. In our hands, disruption of microtubules with nocodazole had no effect on baseline or TNF-α-induced IL-6 or MCP-1 production or secretion from control and TREK-1 deficient AECs.

Interestingly, TREK-1 deficient cells contained increased amounts of α-tubulin at baseline but the significance and the underlying mechanisms for this finding remain to be determined. Currently we can only speculate on how TREK-1 deficiency results in increased α-tubulin levels. We know that in neuronal cells a crosstalk exists between TREK-1 and the F-actin network[45], but whether similar direct interactions exists between TREK-1 and α-tubulin is unknown. We have previously reported that TREK-1 deficiency affected IL-6 mRNA expression[3], and it is possible that TREK-1 similarly affects α-tubulin gene expression. Alternatively, TREK-1 could be a regulatory protein in α-tubulin synthesis. In neurons for example, TREK-1 protein expression can induce the formation of actin- and ezrin-rich membrane protrusions[45], and deficiency of TREK-1 resulted in a decrease in the F-actin content of these cells[46]. Altogether, our data suggest that neither the architecture nor the content of F-actin and α-tubulin filaments play a major role in IL-6 and MCP-1 production and secretion from AECs.

Of particular interest is the fact that although TREK-1 deficient cells contained lower amounts of IL-6 mRNA, the overall fold increase in IL-6 gene expression induced by TNF-α was similar between control and TREK-1 deficient cells when compared to their respective untreated controls. In contrast, MCP-1 mRNA levels increased more in TREK-1 deficient cells that in control cells after TNF-α stimulation. These data give important insight into the regulatory mechanisms underlying IL-6 and MCP-1 production and secretion in TREK-1 deficient AECs. Together with our previously published data showing that the majority of IL-6 and MCP-1 in AECs is newly synthesized[2,3], the results of this study suggest that IL-6 secretion is predominantly regulated post-transcriptionally, whereas MCP-1 secretion is regulated at the transcriptional level. Interestingly, while TREK-1 deficiency resulted in decreased IL-6 and increased MCP-1 secretion from both mouse[1] and human alveolar epithelial cells[2,3], overexpression of TREK-1 had no further effect on MCP-1 secretion when compared to control cells[2].

Particular attention needs to be exerted when interpreting the effects of cytoskeleton altering-agents on mediator release from immune and inflammatory cells. While manipulation of F-actin filaments and microtubules can ultimately result in alterations in inflammatory mediator secretion, many of the studies point out that the underlying mechanisms may be linked to impaired phagocytosis of bacteria and impaired immune cell activation caused by the disruption of cytoskeletal structures rather than by impaired transport of secretory vesicles to the plasma membrane[11,19]. Interestingly, similar findings were observed in lung epithelial A549 cells where inhibition of MCP-1 and IL-8 release was linked to impaired internalization of E. coli bacteria after treatment with cytochalasin D rather than impaired vesicle secretion[20]. Similarly, deceased IL-8 secretion from smoke-exposed A549 cells after cytochalasin D treatment was linked to impaired smoke particle uptake rather than secretory vesicle transport[15]. A further complicating step in understanding these mechanisms is added by the fact that recycling of cytokine and chemokine plasma membrane receptors is generally regulated by cytoskeletal rearrangements[14,47,48]. Therefore, secretion of inflammatory mediators after disruption of cytoskeletal structures could be due to impaired receptor activation and signaling, in addition to defective vesicle secretion.

Unfortunately, our understanding of the secretory mechanisms of lung epithelial cells lags far behind immune and inflammatory cells. Advancing this field is absolutely critical, particularly as epithelial cells emerge as a major contributor to the inflammation observed in ALI/ARDS[49–51]. It appears that the effects of cytoskeletal rearrangements on inflammatory mediator secretion are, at least in part, cell type-specific and can both promote or inhibit cytokine release depending on the activation process, as noted above. We also need further studies targeting the molecular signaling mechanisms that regulate cytoskeletal rearrangements upon epithelial cell activation, including the role of stretch-activated ion channels, such as TREK-1, in epithelial mechano-transduction. Although the gating mechanisms of several ion channels are regulated by cytoskeletal rearrangements[52,53], little is known about the effects of specific ion currents on F-actin and microtubule polymerization and depolymerization. Similarly, in the anterior eye chamber TREK-1 expression caused cytoskeletal changes in response to stretch[54]. Although TREK-1 expression can alter the cytoskeletal structure of various cell types including AECs, we found no effect of such cytoskeletal rearrangements on AEC mediator secretion.

In our hands, we did not observe significant changes in F-actin content or architecture after treatment of control and TREK-1 deficient A549 cells with TNF-α. Nevertheless, exposure of other cell types to TNF-α has been reported to cause significant F-actin rearrangements. For example, cultured pulmonary endothelial cells responded to TNF-α treatment with F-actin thickening and bundling along the cell periphery[55]. It needs to pointed out, however, that the TNF-α dose used in that particular study was four times higher (20 ng/mL) than the dose used in our study (5 ng/mL). In dermal endothelial cells, TNF-α exposure induced an increase in F-actin stress fibers as early as 1–3 hours after treatment, but the dose used was again higher than in our study[56]. A similar F-actin reorganization was observed in immortalized corneal epithelial cells[57] but cells were once again exposed to a higher TNF-α dose (10 ng/mL) and were treated longer (for 24 hours).

To the best of our knowledge, the only study addressing TNF-α-induced F-actin reorganization in lung A549 epithelial cells used 100 ng/ml TNF-α (20-fold higher than our dose) for 48 hours (4 times longer)[58]. In our preliminary studies, such a protocol resulted in significant cell toxicity (data not shown). In another immortalized human adenocarcinoma cell line, H441-4, exposure to TNF-α concentrations up to 100 ng/mL for 48 hours showed no effect on the total actin content, but effects on the F-actin architecture were not reported in that study[59]. We chose a TNF-α dose of 5 ng/mL and an exposure time of up to 6 hours because these conditions induced substantial changes in cytokine gene expression and protein secretion without significant cell toxicity. It is possible that at higher TNF-α doses or after longer exposure periods we would find changes in the F-actin content or architecture in our cells. Furthermore, freshly isolated murine or rat AT II cells may respond to the same treatment conditions differently than cultured human A549 cells. It is, therefore, important to acknowledge that, while TNF-α did not induce cytoskeletal rearrangements in our system, it is well known that it can cause cytoskeletal changes in other cell types[55,57]. However, most of these effects are reported at higher concentrations or longer time periods than the one used in our study.

Limitations of this study include the fact that the experiments were performed in human immortalized cells (A549) rather than in primary cells. However, human A459 cells are a widely accepted model for alveolar epithelial cell biology[60,61]. Furthermore, compared to other alveolar cell lines such as murine MLE-12 cells, human A549 cells secrete higher amounts of inflammatory cytokines and represent a more homogeneous cell population than MLE-12 cells[1,3]. One of the concerns in using a transformed cell line consists in potential non-specific effects caused by the shRNA probe. In preliminary experiments we had confirmed that no non-specific or compensatory changes in the expression of TREK-1-related channels, such as TREK-2 or TRAAK, or in other stretch-activated ion channels such as the Ca^2+^-permeable TRPV4 had occurred (data not shown).

In conclusion, in this study we show for the first time that in TREK-1 deficient AECs alterations in cytokine production and secretion and cytoskeletal derangements occur simultaneously but do not appear to be related. In fact, neither polymerization nor depolymerization of cytoskeletal structures in TREK-1 deficient AECs affected TNF-α-induced IL-6 and MCP-1 gene expression or protein secretion. While the molecular mechanisms underlying these findings require further investigation, TREK-1 remains an important target in the search for new approaches against AEC activation in the context of ALI/ARDS.
